# Development and Validation of a UHPLC-ESI-MS/MS Method for Quantification of Oleandrin and Other Cardiac Glycosides and Evaluation of Their Levels in Herbs and Spices from the Belgian Market

**DOI:** 10.3390/toxins12040243

**Published:** 2020-04-09

**Authors:** Svetlana V. Malysheva, Patrick P. J. Mulder, Julien Masquelier

**Affiliations:** 1 Unit Toxins, Organic Contaminants and Additives, Sciensano, 1050 Brussels, Belgium; julien.masquelier@sciensano.be; 2 Wageningen Food Safety Research, Wageningen University and Research, 6708 WB Wageningen, The Netherlands; patrick.mulder@wur.nl

**Keywords:** oleandrin, LC-MS/MS, plant toxins, validation, herbs, urine

## Abstract

Cardiac glycosides (CGs) are naturally occurring plant secondary metabolites that can be toxic to humans and animals. The aim of this work was to develop a targeted analytical method utilizing liquid chromatography—tandem mass spectrometry (LC-MS/MS) for quantification of these plant toxins in a herbal-based food and human urine. The method included oleandrin, digoxin, digitoxin, convallatoxin, and ouabain. Samples of culinary herbs were extracted with acetonitrile and cleaned using Oasis^®^ MAX solid-phase extraction (SPE), while samples of urine were diluted with acidified water and purified on Oasis^®^ HLB SPE cartridges. Limits of quantification were in the range of 1.5–15 ng/g for herbs and 0.025–1 ng/mL for urine. The mean recovery of the method complied with the acceptable range of 70–120% for most CGs, and relative standard deviations were at maximum 14% and 19% for repeatability and reproducibility, respectively. Method linearity was good with calculated R² values above 0.997. The expanded measurement uncertainty was estimated to be in the range of 7–37%. The LC-MS/MS method was used to examine 65 samples of culinary herbs and herb and spice mixtures collected in Belgium, from supermarkets and local stores. The samples were found to be free from the analyzed CGs.

## 1. Introduction

**Figure 1:**
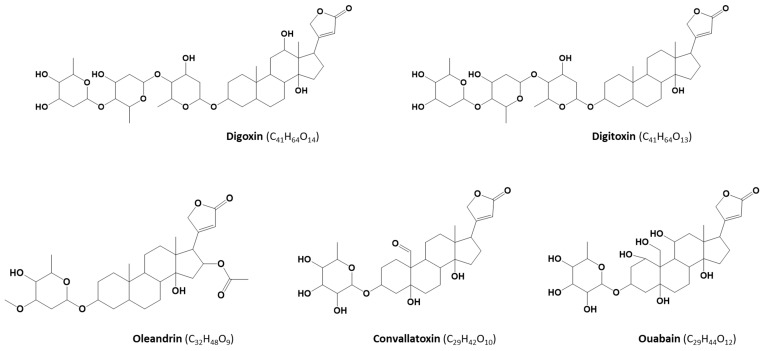
Chemical structures of the target cardiac glycosides.

Cardiac glycosides (CGs) are secondary metabolites produced by plants belonging to, among others, the genera *Nerium* (oleander)*, Convallaria* (lily-of-the-valley), and *Digitalis* (foxglove). The core structure of most CGs consists of lactone and steroid rings and a sugar moiety (Figure 1). CGs occur in all parts of plants and can be poisonous to livestock and humans. Their primary mechanism of action is inhibition of the membrane sodium-potassium pump that influences the intracellular sodium, calcium, and potassium concentrations and, as a consequence, causes disruptions in the cardiovascular system. However, other symptoms of toxicity may also include gastrointestinal, ocular, and neurologic disorders. In a specific dose range, however, CGs such as digoxin (DIGO) and digitoxin (DIGI) (Figure 1), have a long history of use as medications in treating various heart conditions [1,2,3,4,5,6].

In the literature, numerous cases of human poisoning with plants containing CGs through self-medication, accidental ingestion, suicide attempts, or criminal administration have been documented [7,8,9,10,11]. These reports also included a remarkable case of intoxication with CGs through food [8]. Superficial resemblance of the leaves of *Nerium oleander*, a plant producing toxic CG oleandrin (OLE), to the leaves of olive and bay trees (*Laurus nobilis*) might contribute to misidentification of the plant material and to accidental poisoning. Renal excretion is the main elimination route for some CGs (e.g., DIGO), while the hepatic route is more common to other CGs, combined with a partial renal elimination [12]. CGs excreted in urine are mainly unchanged [13,14,15] or partially metabolized [16].

A number of analytical, mostly single-analyte, methods for quantification of CGs in biological matrices have been described [11,17,18,19,20,21,22,23,24]. As a detection technique, these methods utilized mass spectrometry (MS) coupled to liquid chromatography (LC), which, thanks to its good selectivity and sensitivity, has nowadays become the method of choice for many applications including toxin analysis. Other techniques, such as immunoassay, (high-performance) thin-layer chromatography, and high-performance LC coupled to a UV or fluorescence detector [25,26,27,28,29], have also been applied to the detection and quantification of CGs.

Analytical methods for determination of these plant toxins in other than clinical samples are currently scarce. However, in case of a poisoning incident originating from the alimentary chain, the availability of a reliable method for food products is essential to confirm or rule out the ingestion of CG-containing plant material. Owing to the frequent use of herbal products as composite blends, and the complexity of the CG compound class, more than one CG might be associated with the poisoning, which points towards the significance of setting up multi-analyte methods. Therefore, the objective of this work was the development and validation of an ultra-high-performance (UHPLC)-MS/MS method for quantification of five plant toxins, namely OLE, DIGO, DIGI, convallatoxin (CON), and ouabain (OUB) (Figure 1), in edible herbs and spices and, complementary, in human urine. The choice of the target glycosides was dictated by their toxicity, known intoxication cases, occurrence of CG-producing plants, and availability of commercial reference standards.

## 2. Results

### 2.1. Optimization of LC-MS/MS Conditions

**Figure 2:**
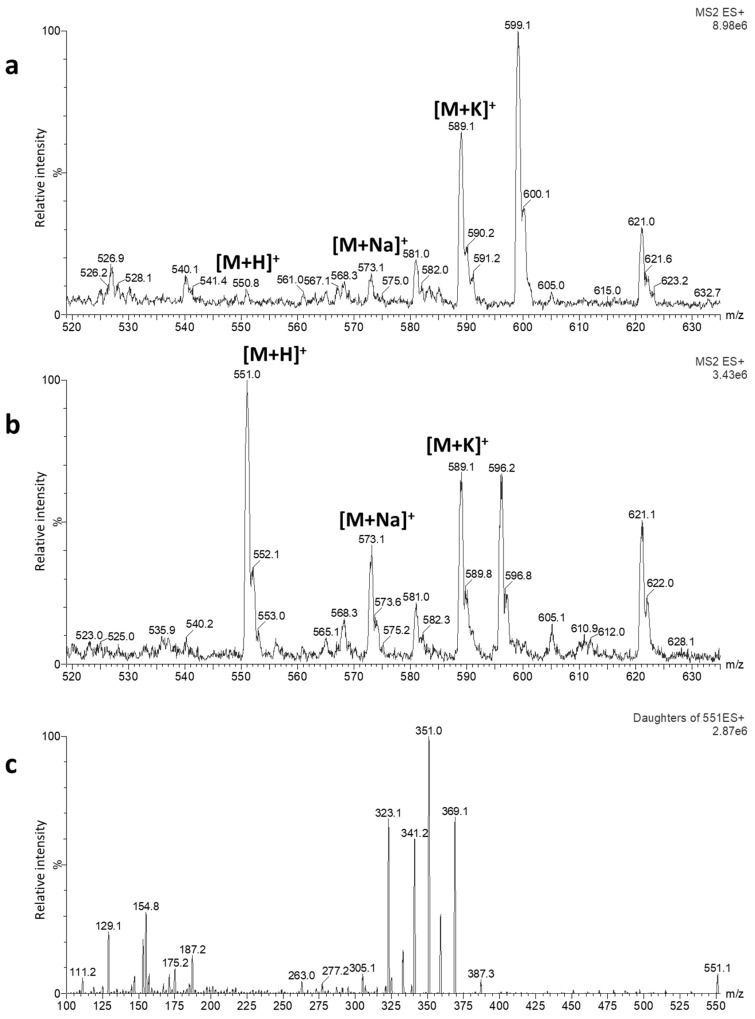
Full ESI(+)-MS spectra obtained through the flow injection analysis of a 1 µg/mL solution of convallatoxin (CON) in H_2_O + 10 mM HCOONH_4_ (pH 3):acetonitrile (ACN) (50:50, v/v) (**a**) and H_2_O + 10 mM NH_4_HCO_3_ (pH 9):ACN (50:50, v/v) (**b**) and ESI(+)-MS/MS spectrum in H_2_O + 10 mM NH_4_HCO_3_ (pH 9):ACN (50:50, v/v) (**c**). The vertical axes represent relative peak intensity (normalized to 100%), while the horizontal axes display measured *m/z* (mass-to-charge ratio) values. The MS setup is given in Section 5.3.

In this study, basic (pH 9, 10 mM ammonium bicarbonate (NH_4_HCO_3_) with ammonia (NH_3_)) and acidic (pH 3, 10 mM ammonium formate (HCOONH_4_) with formic acid (HCOOH)) aqueous mobile phases in combination with acetonitrile (ACN) as an organic phase were used to optimize the MS ionization of target CGs. As the initial step of optimization, the flow injection analysis was performed in electrospray ionization positive (ESI(+)) and negative (ESI(-)) modes. In the acidic mobile phase, the presence of intense [M + Na]^+^ and [M + K]^+^ adducts, which did not further fragment, was observed for all CGs; therefore, further optimizations were carried out with the basic mobile phase. Figure 2a,b demonstrates a full ESI(+)-MS spectrum of CON in the mobile phase at pH 3, in which an abundant presence of [M + Na]^+^ and [M + K]^+^ adducts can be clearly observed, and a full ESI(+)-MS spectrum in the basic mobile phase, in which the abundant presence of the molecular ion is apparent. As opposed to [M + Na]^+^ and [M + K]^+^ ions, fragmentation of the molecular ion provided several abundant product ions (Figure 2c), usable for defining selected reaction monitoring (SRM) transitions. While in the ESI(+) mode [M + H]^+^ or [M + NH_4_]^+^ ions were abundant in the spectrum, the full MS scan in the ESI(-) mode revealed sufficient abundance of [M-H]^-^ ions for all CGs. The final MS and MS/MS conditions (Table 1) were optimized in ESI(+), because of the higher intensity of MS signals in this mode.

**Figure 3:**
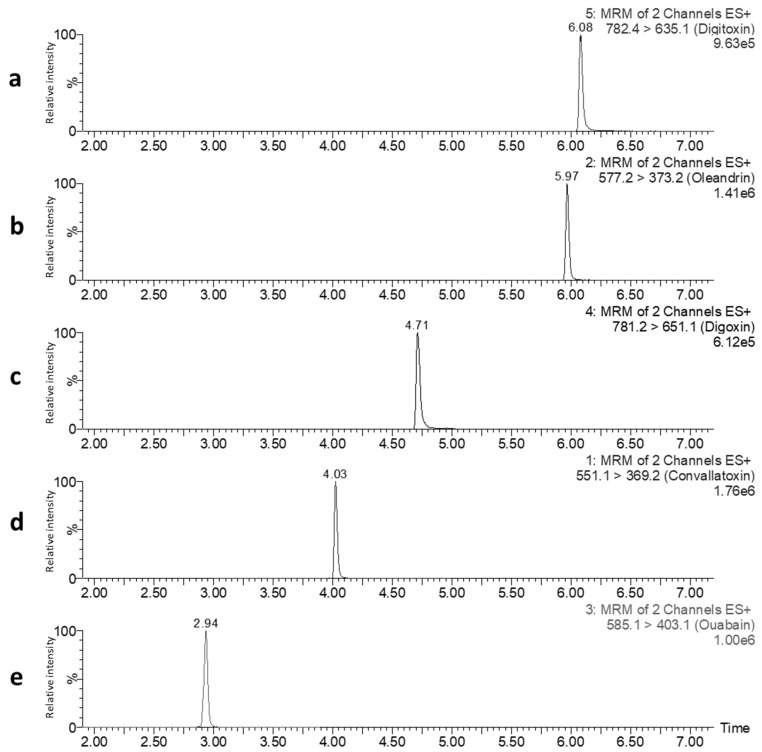
LC-MS/MS selected reaction monitoring (SRM) chromatograms of a single injection of a standard mixture of DIGI (**a**), DIGO (**c**), CON (**d**), and ouabain (OUB) (**e**) at a concentration of 2.5 ng/mL and OLE (**b**) at a concentration of 0.25 ng/mL, dissolved in H_2_O:ACN (80:20, v/v). For each cardiac glycoside (CG) the most abundant SRM transition is displayed. The vertical axes represent relative peak intensity (normalized to 100%), while the horizontal axes display retention time (in min). The chromatographic conditions applied are given in Section 5.2.

For LC separation of the target analytes, the suitability of a UHPLC column with the C18 BEH stationary phase in combination with the basic mobile phase consisting of ACN and 10 mM NH_4_HCO_3_ at pH 9, was tested. Using a gradient elution (see Section 5.2), good separation of the CGs was achieved (Figure 3).

### 2.2. Optimization of Sample Preparation

For the evaluation of extraction and clean-up recovery, analyte-free herbal and urine samples spiked with the target CGs before and after extraction and/or clean-up step were prepared.

A herbal mixture (herbes de Provence) was used as a test mixture for the extraction experiments. ACN, methanol (MeOH), and H_2_O, as single solvents or as mixtures, were tested. It was found that good extraction recoveries (>70%) were obtained with ACN, MeOH, ACN:H_2_O (50:50, v/v), and MeOH:H_2_O (50:50, v/v), with slightly better results for OUB if MeOH was used in the extraction solvent. This method was aimed at achieving as low as possible limits of quantification (LOQs). It was apparent that to accomplish that a further clean-up and concentration step of the extract was necessary. The widely-used QuEChERS method [30], which combines extraction of a sample with ACN, salting-out, and subsequent dispersive solid phase extraction (SPE) clean-up, was tried, however, it demonstrated low extraction recoveries and poor clean-up efficiency. ENVI-Carb™ SPE with a graphite sorbent, which is very suitable for elimination of pigments that are abundantly present in herbs, resulted in no recovery of the target CGs. Other SPE cartridges, such as Discovery^®^ DSC-18, and Oasis^®^ HLB, provided acceptable recoveries but matrix effects were pronounced, affecting the sensitivity of the method. Oasis^®^ MAX SPE was found to be the most suitable for clean-up of the herb samples, as it showed reduced matrix effects, good recoveries, and improved estimated LOQs. Among the tested extraction solvents, ACN was best compatible with the required setup of the Oasis^®^ MAX protocol. Other solvents in combination with the herb matrix caused blockage of SPE cartridges or of the filter prior to the SPE. It should be mentioned that none of the tested protocols provided a sufficiently low LOQ and reproducible results for OUB, therefore, this compound was not included in the final method for herbal samples.

The sample preparation for urine was more straightforward and consisted of a sample dilution with H_2_O containing 2% HCOOH and clean-up with Oasis^®^ HLB SPE. The subsequent extract concentration was necessary to achieve a higher method sensitivity. In urine, this protocol was able to provide reproducible results for OUB and thus all selected plant toxins were included in the final method for this matrix.

The detailed protocols for extraction and clean-up of herb and urine samples are given in Section 5.4.

### 2.3. Method Validation

The method validation data for herbs and urine are summarized in Table 2 and Table 3, respectively. The method LOQs were calculated based on a signal-to-noise ratio (S/N) approach and are reported in order to simplify the comparison with other methods for CGs described in the literature. The LOQs were in the range from 1.5 to 15 ng/g for herbs and from 0.025 to 1 ng/mL for urine, with OLE showing the highest sensitivity among the target CGs (Table S1). The analysis of blank herb and urine samples demonstrated that no peak with a S/N of at least 3 was detected at the expected retention time of the CGs, pointing out good specificity of the method. The matrix effect experiments revealed that the calculated *t*-value for the target CGs in herbs and urine were much greater than the tabulated *t*-value at the 95% confidence level indicating a significant difference between the slopes of calibration curves in the solvent and matrix, i.e., the presence of matrix effects. All CGs in both matrices suffered from a signal suppression, with the strongest suppression for OUB and the smallest effect for OLE and DIGI. The calibration curves were prepared in matrix extracts (spiked post clean-up) by plotting the concentration of the analyte in the calibration standards against the ratio of peak area of the analyte to the internal standard, digoxin-d_3_ (DIGO-D), for all target CGs. Calibration curves for herb and urine samples were linear over the validated concentration range with coefficients of determination (R^2^) >0.997. The lowest calibration level (LCL) is used as a reporting limit for quantification of CGs in herbs and urine. The mean (apparent) recovery data obtained for three concentration levels in herbs were in the range from 83% to 115% for OLE, DIGO, and DIGI, and 55% for CON. The mean recoveries for all CGs in urine ranged from 80% to 96%. The method precision was expressed as a relative standard deviation (RSD) of replicate measurements. For herbs, the repeatability (RSD_r_) of the method ranged from 6% to 14% and the within-laboratory reproducibility (RSD_wR_) was from 7% to 17%, while these parameters for urine ranged from 1% to 7% and from 5% to 19%, respectively. The expanded measurement uncertainty (MU) was not higher than 28% and 37% at the lowest concentration levels validated for herbs and urine, respectively. The uncertainty at higher concentration levels did not exceed 31% and 16% for herbs and urine, respectively.

### 2.4. Method Application for Analysis of Culinary Herbs

The validated LC-MS/MS method was subsequently used to investigate the contamination of culinary herbs and spices that are available on the Belgian food market. In total, 65 samples were acquired in supermarkets and organic food shops and comprised the culinary herbs and herb/spice mixtures containing bay leaves (*Laurus nobilis*). For the majority of samples, the country of production was not specified. About 20% of samples originated from organic farming. The detailed information on ingredients of the samples is given in Table S2.

Quality control samples, namely a standard mixture of CGs in a neat solvent and herb mixture fortified with CGs at the concentrations corresponding to the middle validated level, were included in each sample sequence. Identification of CGs in samples was completed following the Commission Decision 2002/657/EC [31]. This implied the presence of a peak of the target analyte with a S/N ratio of at least 3 for each ion transition, compliance of relative retention times, and conformity of deviations of relative ion intensities with regards to the matrix-matched calibration standards. The most abundant product ion was used for quantification, while the second product ion was used for confirmation of the analytes. The analysis demonstrated that none of the collected samples contained CGs above the LCL.

## 3. Discussion

Phytotherapeutic and nutritional use of plants and herbal-based products has (re)gained its popularity in the last years. Due to improper usage of plants or unawareness of plant toxicity, several intoxication cases with CG-containing plants have been reported recently [7,10,11]. Ingestion of toxic plant (parts) may also be possible through the food chain. Plant misidentification and inadequate control of harvesting or processing may lead to an unintentional mix of toxic plant material with the raw plant material used for production. Since more analytical methods became available for screening of organic molecules at low levels, several contaminants have surfaced as an issue of relevance in food safety. That was the case for other plant toxins, such as tropane alkaloids and pyrrolizidine alkaloids, which were found in herbal teas, herbs and spices, cereal-based food, and herbal food supplements [32,33,34,35], sometimes at levels that can represent risk for human health [36,37]. The current study aimed at developing a reliable analytical method for detection of CGs in plant-based food products. As a complementary tool for control of poisoning incidents, the method was also validated for the urine matrix. As opposed to some other human biological fluids that are used to study exposure to contaminants, urine is easily accessible from individuals of all ages, can be obtained in larger volumes, and its collection method is noninvasive.

As a detection technique, LC-MS/MS was chosen for this work, as it allows a high-throughput simultaneous detection of structurally diverse molecules, including compounds of natural origin, at trace levels and with high selectivity and specificity. In the proposed method, the SRM mode was used to obtain increased sensitivity and specificity, the parameters that are of great importance in the analysis of such complex matrices as herbs and urine. The selected ionization mode was ESI, the commonly used interface in the LC-MS analysis of natural toxins. Though less frequent, atmospheric pressure chemical ionization (APCI) is also used for the ionization of small molecules. Sugergat et al. [38] compared these two ionization modes for the analysis of DIGO in human serum and observed a lower intensity of the protonated molecule and a higher degree of fragmentation, resulting in lower sensitivity of the APCI mode compared to ESI.

For some molecules, formation of alkali metal adducts can be observed in ESI-MS. This can possibly be attributed to leaching from glass recipients or the presence of impurities in the mobile phase [39]. Such adducts, as compared to the molecular ions, can be unstable and might not produce fragment ions, jeopardizing the reliability of a quantitative LC-MS/MS method. The particular issue of metal adduct formation has also been reported for CGs [18]. In this study, formation of metal adducts, not prone to fragmentation, was observed when a mobile phase with HCOONH_4_ and HCOOH (pH 3) was used for the LC. In order to generate a sensitive single precursor ion for the analysis in the SRM mode, Bylda et al. investigated different mobile phase additives and finally selected the [M + Li]^+^ adduct for the quantification of CGs [18]. In the absence of intense molecular ions, some applications used [M + Na]^+^ or [M + K]^+^ adducts for quantification of CGs in a single or selected ion monitoring (SIM) mode [38,40]. However, it has been noticed that SIM produced much higher detection limits with biological samples compared to SRM, while the two modes were similar in sensitivity if a standard mixture of CGs containing no matrix was injected [21]. In the current method, under the applied LC-MS conditions (see Section 5.2) with a NH_4_HCO_3_-containing mobile phase (pH 9), formation of a protonated molecular ion [M + H]^+^ for DIGO, OLE, OUB, and CON, and of an [M + NH_4_]^+^ adduct for DIGI was achieved. Under mild fragmentation conditions, these precursor ions underwent collision-induced dissociation in the quadrupole yielding usable intense product ions. Of these fragments, the two most abundant ions were selected for the identification and quantification of these plant toxins, thereby fulfilling the requirements of the Commission Decision 2002/657/EC, which recommends the use of four identification points for confident identification of compounds in the LC-MS/MS analysis [31]. Some of the observed abundant product ions corresponded to sequential losses of the sugar moieties and elimination of hydroxyl groups from the steroid aglycones [41].

To optimize the sensitivity and selectivity of the LC-MS/MS method, SPE was used in the sample preparation of CGs in herbs and urine, in order to reduce complexity of the matrix and to allow enrichment of the analytes. This approach has previously been used for purification of some CGs in whole blood and plasma [17,24,42]. Compared to Oasis^®^ HLB SPE devices, used for the clean-up of urine samples in the current method, a significant reduction of matrix effects for herb samples was noted with Oasis^®^ MAX cartridges.

Currently, no maximum levels for CGs in food are set and analytical methods should preferably achieve as low as possible LOQs. The results of validation indicated that the developed method is able to detect and quantify the target CGs at low levels. In comparison to other existing methods for CGs in biological matrices and herbal-based products (Table S1), the current method is able to reach lower or similar LOQs. During the validation, matrix effects were observed for herbs and urine. The matrix effect is in many occasions unavoidable in MS analysis of complex matrices. It could be caused by co-eluting compounds that interfere with the ionization process of the target analytes leading to signal suppression or enhancement. The CGs were differently affected by the matrix interferences. OUB, being the most polar CG and eluting early in the chromatographic run, suffered from a greater matrix suppression than OLE and DIGI, which eluted at the end of the run. This can be associated with the fact that hydrophilic compounds of the biological or plant matrix also eluted early in the chromatographic run giving rise to a more pronounced matrix effect in this region of the chromatogram. The addition of stable isotope labeled analogues of the analyzed molecules is suggested for use as internal standards to counteract the matrix effects. The use of commercially available or in-house synthesized labeled internal standards have already been reported for the LC-MS analysis of a number of CGs [18,19]. Other structurally related molecules, such as methyldigoxin, digitoxigenin, and gitoxigenin, were included as internal standards in some analytical methods [21,40,43]. In the current method DIGO-D, the deuterated analogue of DIGO, was applied as an internal standard for all target CGs.

The method linearity was tested and found to be good exhibiting R^2^ >0.997, in most cases >0.999. The mean recovery of the developed method was in agreement with the acceptable limit of 70–120%, except for CON in herbs. A good precision of the method was demonstrated with repeatability and within-laboratory reproducibility below 20% for both matrices. The accuracy and the precision of the developed method were in the same range as for other methods, reported in the literature, for quantification of CGs in biological matrices (Table S1). The obtained MU was below 50%, complying with the SANTE/12682/2019 guidance document [44].

After completing the validation, the developed LC-MS/MS method was applied, to assess the CG contamination of herbs and herbal/spice mixtures used for culinary purposes, available on the Belgian food market. None of the target CGs were detected in these samples indicating that there is currently no safety risk for the population with regards to contamination of culinary herbs and blends with CGs.

CGs are a large and diverse group of naturally occurring toxic compounds, and, upon necessity, the LC-MS technique allows extension of the method to other plant toxins from this class. The presented method can, on the one hand, be used in food control initiatives to ensure food safety and, on the other hand, in population-wide survey studies that combine monitoring of food contamination and analysis of human biological fluids to unravel the level of exposure to plant toxins.

## 4. Conclusions

This study describes the first detailed validated method for quantification of OLE and other CGs in culinary herbs and human urine. The method displays good specificity, linearity, accuracy, and expanded measurement uncertainty, thus enabling the accurate quantification of OLE, DIGO, DIGI, and CON in culinary herbs and OLE, DIGO, DIGI, CON, and OUB in human urine. This new method was applied to the analysis of more than 60 samples of culinary herbs and herb/spice mixtures containing bay leaves present on the Belgian food market, showing that these products are safe for the consumer. The UHPLC-ESI-MS/MS method described here could, therefore, become a useful tool to determine these plant toxins in culinary herbs and also in urine.

## 5. Materials and Methods

### 5.1. Standards, Reagents, and Consumables

Analytical standards of OLE, DIGO, DIGI, CON, and OUB octahydrate were purchased from Sigma-Aldrich (Buchs, Switzerland). Individual stock solutions were prepared by dissolving the crystalline standards in MeOH at a concentration of 1 mg/mL. A methanolic solution of DIGO-D (1 mg/mL) was obtained from Cayman Chemicals (Ann Arbor, MI, USA). Intermediate solutions were prepared by diluting the stock solutions in MeOH. The stock and the intermediate solutions were stored at −20 °C.

The MeOH absolute ULC-MS, ACN ULC-MS, and HCOOH 99% ULC-MS were purchased from Biosolve (Valkenswaard, the Netherlands). The ammonia solution 28–30% was obtained from Merck (Darmstadt, Germany), while NH_4_HCO_3_ LC-MS and HCOONH_4_ LC-MS were supplied by Sigma-Aldrich (Steinheim, Germany). H_2_O was purified by a Milli-Q purification system (Millipore Corp., Bedford, MA, USA).

Oasis^®^ MAX (3 cc, 60 mg) LP and Oasis^®^ HLB (3 cc, 60 mg) extraction cartridges were provided by Waters (Wexford, Ireland). Discovery^®^ DSC-18 (6 mL, 500 mg) and Supelclean™ ENVI-Carb™ (6 mL, 500 mg) SPE cartridges were obtained from Sigma-Aldrich. VWR (Randor, PA, USA) was the supplier of 15 and 50 mL centrifuge PP tubes and centrifugal filters (modified nylon, 0.2 µm, 500 µL).

### 5.2. UHPLC-MS/MS Conditions

The UHPLC-MS/MS system consisted of an ACQUITY UPLC H-class system coupled to a Xevo TQ-S triple quadrupole mass spectrometer (Waters, Milford, MA, USA).

The mass spectrometer was operated in the ESI(+) mode. The MS parameters were set as follows: Source and desolvation temperatures: 150 and 350 °C, respectively; capillary voltage: 1.50 kV; cone and desolvation gas flows: 150 and 1000 L/h, respectively; collision gas flow: 0.15 mL/min; source offset: 30 V. The SRM acquisition mode was used.

Chromatographic separation was achieved on an AQUITY UPLC^TM^ BEH C18 column (2.1 × 100 mm; 1.7 µm) with an ACQUITY UPLC^TM^ BEH C18 VanGuard precolumn (2.1 × 5 mm; 1.7 µm) (both from Waters). The column temperature was maintained at 40 °C. The mobile phase was composed of phase A (H_2_O containing 10 mM NH_4_HCO_3_ pH 9) and phase B (ACN). The flow rate used was 0.45 mL/min and the applied gradient elution program was as follows: 0–1 min: 95% A, 1–6 min: 40% A, 6–7 min: 10% A, 7–7.1 min: 95% A, 7.1–10 min: 95% A. The injection volume was 10 µL.

### 5.3. Samples

The samples of culinary herbs were purchased from supermarkets and local (organic food) stores in Belgium and included 14 single herbs and 51 herb/spice mixtures. More details on the samples are provided in Table S2. Prior to the analysis, the herb samples were finely ground and homogenized. The samples of human urine were provided by volunteers.

### 5.4. Sample Preparation

Two grams of the herb sample was weighed in a 50 mL PP tube. After addition of 25 mL of ACN, the sample was vigorously shaken on an overhead shaker for 30 min and centrifuged for 10 min at 3180× *g*. Ten mL of supernatant was transferred in a 15 mL PP tube and evaporated at 45 °C under a stream of nitrogen until a volume of approximately 1 mL. Subsequently, H_2_O containing 5% NH_3_ was added to a total volume of 10 mL. After thorough vortexing and centrifugation (10 min at 3180× *g*) the extract was subjected to further clean-up. Oasis^®^ MAX SPE cartridges were conditioned with 3 mL MeOH and 3 mL H_2_O. Three mL of supernatant was loaded onto the cartridge and washed with 3 mL H_2_O containing 5% NH_3_. After a brief drying step, the target analytes were eluted with 3 mL MeOH and collected in 15 mL PP tubes. The eluate was evaporated until dryness at 45 °C under a stream of nitrogen. The residue was reconstituted in 250 µL H_2_O:ACN (80:20, v/v) and filtered through filter units for 5 min at 14,000× *g*.

Five mL of urine was transferred to a 15 mL PP tube, to which 5 mL H_2_O containing 2% HCOOH was added. After vortexing, the sample was centrifuged for 10 min at 3180× *g*. For the clean-up, Oasis^®^ HLB cartridges were conditioned with 3 mL MeOH and 3 mL H_2_O. Six mL of supernatant was loaded onto the cartridge and washed with 3 mL H_2_O. After a brief drying step, the target analytes were eluted with 3 mL MeOH and collected in 15 mL PP tubes. The eluate was evaporated until dryness at 45 °C under a stream of nitrogen. The residue was reconstituted in 500 µL H_2_O:ACN (80:20, v/v) and filtered through filter units for 5 min at 14,000× *g*.

### 5.5. Validation

The validation study was performed using spiked analyte-free representative sample materials. The following method parameters were evaluated: LOQ, specificity, linearity, recovery, repeatability (RSD_r_), reproducibility (RSD_wR_), matrix effects, and expanded measurement uncertainty (MU).

LOQ was defined as the minimum analyte concentration in the spiked samples that produced an SRM transition with a minimum S/N of 10. Specificity of the method was assessed through the analysis of blank matrix samples. The absence of a peak with a S/N ≥ 3 at the expected retention time of the target CG indicated good method specificity. Linearity of the method was evaluated by fortifying blank matrix samples with the target analytes at varying concentrations (minimum five levels). A logarithmic transformation of the axes and a linear regression model were applied. (Apparent) recovery was assessed by a spiking of blank matrix with the target analytes at three concentration levels in triplicate. The measured concentrations were determined using a matrix-matched calibration curve and the recovery was calculated as follows (Equation 1):

Recovery (%) = Measured concentration/Theoretical concentration × 100.(1)

For determination of the repeatability (RSD_r_), spiking experiments were performed at three concentration levels in triplicate on the same day, while for within-laboratory reproducibility (RSD_wR_) evaluation, the same experiments were carried out on three days. Matrix effects were assessed by comparing the slopes of calibration curves prepared in the matrix extract and neat solvent. The *t*-test was used for statistical evaluation of the matrix effect data. The expanded measurement uncertainty (MU) (corresponding to a 95% confidence level and a coverage factor of 2) was estimated according to [44].

## Figures and Tables

**Table 1:** Electrospray ionization positive (ESI)(+)-MS/MS parameters for detection of cardiac glycosides.

Analyte	Precursor Ion (*m/z*)	Cone Voltage (V)	Product Ions (*m/z*)	Collision Energy (eV)
Oleandrin	577.2 [M + H]^+^	30	**373.2**^1^ 433.1	15 10
Digoxin	781.2 [M + H]^+^	25	**651.1** 391.1	10 15
Digitoxin	782.4 [M + NH_4_]^+^	30	**635.1** 375.1	10 20
Convallatoxin	551.1 [M + H]^+^	20	**369.2** 351.2	10 20
Ouabain	585.1 [M + H]^+^	30	**403.1** 385.1	15 20
Digoxin-d_3_^2^	784.2 [M + H]^+^	25	**654.1** 394.2	10 15

^1^ Highlighted in bold: Most abundant product ion, ^2^ internal standard.

**Table 2:** Validation data for cardiac glycosides in culinary herbs.

Analyte	Reporting Limit (ng/g)	Linear Range (ng/g)	R^2^	Concentration Level	Recovery ± SD^1^	Repeatability (RSD_r_) (%)	Reproducibility (RSD_wR_) (%)	Measurement Uncertainty
				(ng/g)	(%)			(%)
Oleandrin	2.5	2.5–200	0.9993	5	82 ± 7	7	8	17
				25	86 ± 6	6	7	14
				100	80 ± 6	6	7	15
					Mean: 83 ± 6	Mean: 6	Mean: 8	
Digoxin	10	10–400	0.9995	10	121 ± 11	11	11	21
				50	114 ± 7	7	7	13
				200	111 ± 10	6	10	19
					Mean: 115 ± 10	Mean: 9	Mean: 9	
Digitoxin	20	20–400	0.9995	20	86 ± 10	14	14	27
				100	96 ± 11	9	12	24
				400	95 ± 13	7	15	31
					Mean: 92 ± 12	Mean: 10	Mean: 14	
Convallatoxin	10	10–400	0.9994	10	52 ± 7	7	14	28
				50	55 ± 9	12	17	34
				200	57 ± 7	10	12	25
					Mean: 55 ± 7	Mean: 9	Mean: 14	

^1^ SD: Standard deviation.

**Table 3:** Validation data for cardiac glycosides in human urine.

Analyte	Reporting Limit (ng/mL)	Linear Range (ng/mL)	R^2^	Concentration Level	Recovery ± SD^1^	Repeatability (RSD_r_) (%)	Reproducibility (RSD_wR_) (%)	Measurement Uncertainty
				(ng/mL)	(%)			(%)
Oleandrin	0.1	0.1–20	0.9987	0.5	80 ± 13	3	18	36
				2.5	84 ± 9	3	13	26
				10	87 ± 5	2	7	13
					Mean: 84 ± 10	Mean: 3	Mean: 13	
Digoxin	1	1–200	0.9993	5	93 ± 10	4	12	24
				25	92 ± 6	2	7	14
				100	92 ± 4	3	5	9
					Mean: 92 ± 7	Mean: 3	Mean: 8	
Digitoxin	1	1–200	0.9975	5	86 ± 14	7	19	37
				25	95 ± 10	6	12	24
				100	88 ± 5	3	5	11
					Mean: 89 ± 11	Mean: 5	Mean: 12	
Convallatoxin	1	1–200	0.9993	5	91 ± 15	4	19	36
				25	94 ± 7	2	8	26
				100	96 ± 3	1	3	16
					Mean: 94 ± 9	Mean: 3	Mean: 10	
Ouabain	1	1–200	0.9994	5	87 ± 14	4	18	37
				25	89 ± 11	6	13	17
				100	93 ± 7	4	8	7
					Mean: 90 ± 11	Mean: 5	Mean: 13	

^1^ SD: Standard deviation.

## Supplementary Materials

The following are available online at https://www.mdpi.com/2072-6651/12/4/243/s1, Table S1: Comparison of performance characteristics of different LC-MS/MS methods for analysis of cardiac glycosides; Table S2: Overview of culinary herbs and spices collected in Belgian food stores.
