# Supplementary material for: Development and Validation of a UHPLC-ESI-MS/MS Method for Quantification of Oleandrin and Other Cardiac Glycosides and Evaluation of Their Levels in Herbs and Spices from the Belgian Market
Source: Toxins (Basel). 2020 Apr 9;12(4):243. doi: 10.3390/toxins12040243 (PMC7232191; doi:10.3390/toxins12040243)
Supplement: Supplementary file 1 [file toxins-12-00243-s001.pdf]

Supplementary Materials: Development and Validation of a UHPLC-ESI-MS/MS Method for Quantification of Oleandrin and Other Cardiac Glycosides and Evaluation of Their Levels in Herbs and Spices from the Belgian Market

Svetlana V. Malysheva *, Patrick P. J. Mulder and Julien Masquelier

Table S1. Comparison of performance characteristics of different LC-MS/MS methods for analysis of cardiac glycosides.

Cardiac Glycoside(s)	Matrix	Method	Limit of Quantification	Recovery (%)[3]	Repeatability (%)	Reproducibility (%)	Reference[5]
Oleandrin	culinary herbs	UHPLC-MS/MS	1.5 ng/g	83	6	8	current study
Digoxin			7.5 ng/g	115	9	9	
Digitoxin			15 ng/g	92	10	14	
Convallatoxin			7.5 ng/g	55	9	14	
Oleandrin	human urine	UHPLC-MS/MS	0.025 ng/mL	84	3	13	current study
Digoxin			0.5 ng/mL	92	3	8	
Digitoxin			0.25 ng/mL	89	5	12	
Convallatoxin			1 ng/mL	94	3	10	
Ouabain			1 ng/mL	90	5	13	
Digoxin	human serum	HPLC-MS/MS	0.12 ng/mL	96	6	9	[1]
Digitoxin			0.4 ng/mL	91	11	14	
Digoxin	human whole blood	HPLC-MS/MS	0.15 ng/mL[1]	106	7	/	[2]
Digitoxin			0.3 ng/mL[1]	113	10		
Digoxin	human urine		0.15 ng/mL[1]	94	5		
Digitoxin			0.3 ng/mL[1]	89	9		
Oleandrin	bovine serum	UHPLC-MS/MS	0.36 ng/mL	63	/	/	[3]
	bovine blood			78			
	bovine heart			80			
	bovine liver			80			
Oleandrin	human plasma	HPLC-MS/MS	3 ng/mL[1]	/[4]	/	/	[4]
Oleandrin	human whole blood	HPLC-MS/MS	0.023 ng/mL[2]	>92	<15	<15	[5]

Cardiac Glycoside(s)	Matrix	Method	Limit of Quantification	Recovery (%)[3]	Repeatability (%)	Reproducibility (%)	Reference[5]
Oleandrin	herbal cosmetics	UHPLC-MS/MS	5 ng/g	98	5	5	[6]
Digoxin	culture medium	HPLC-MS/MS	0.5 ng/mL	99	5	5	[7]
Ouabain				101	3	4	
Digoxin	cell lysate			101	8	7	
Ouabain				105	4	7	
Digoxin	human serum	HPLC-MS/MS	0.1 ng/mL	101	6	8	[8]
Oleandrin	human blood	LC-MS/MS	3 ng/mL[1]	98	<11	<11	[9]
	human liver		6 ng/g[1]	112			
Digoxin	human whole blood	UHPLC-MS/MS	0.28 ng/mL	104	6	6	[10]
Convallatoxin	human whole blood	UHPLC-MS/MS	4.2 ng/mL[1]	111	13	13	[11]
Digitoxin			0.3 ng/mL[1]	102	14	14	
Digoxin			0.6 ng/mL[1]	98	7	9	
Oleandrin			0.3 ng/mL[1]	96	11	15	

[1] estimated from the reported limits of detection using a factor of 3; [2] estimated from the lowest reported value with regards to S/N of 10; [3] a mean value across different validated levels, where applicable; [4] not reported or not evaluated; [5] a list of references is provided at the end of Supplementary Materials.

Table S2. Overview of culinary herbs and spices collected in Belgian food stores.

Sample Code	Product Name	Farming	Country of Production	Ingredients
Sample 1	Garam masala spice mix	non-organic	Germany	coriander (30%), black pepper (11%), cumin, garlic (10%), chili pepper (5.5%), cinnamon (4%), clove (3.5%), ginger, bay leaf (3%)
Sample 2	Italian herb mix	organic	n.s.[1]	mild chili, oregano, thyme, bay leaf, sage, ground coriander seeds, basil, black pepper
Sample 3	Mix for pasta	organic	n.s.	wild marjoram, thyme, basil, rosemary, garlic, onion, savory, bay leaf
Sample 4	Herb mix for fish	organic	n.s.	fennel, thyme, parsley, bay leaf
Sample 5	Bay leaves	organic	Turkey	bay leaves
Sample 6	Garam masala spice mix	non-organic	France	coriander seeds (49%), black pepper, Jamaican pepper, cumin (6%), cardamom (6%), turmeric, nutmeg, ginger, clove, bay leaf, cinnamon, chili powder, cayenne pepper

Sample Code	Product Name	Farming	Country of Production	Ingredients
Sample 7	Mix for beef stew	non-organic	France	dried onion (36%), bell pepper (6%), coriander seed, mustard seed (4%), Jamaican pepper, ginger, white pepper, nutmeg, bay leaf, thyme
Sample 8	Taste refiner	non-organic	n.s.	onion, garlic, turmeric, celery seed, clove, bay leaf
Sample 9	Bouquet garni	non-organic	n.s.	bay leaf, thyme
Sample 10	Bay leaves	non-organic	n.s.	bay leaves
Sample 11	Mix for mussels	non-organic	n.s.	vegetables, chives, parsley, tarragon, dill, bay leaf, thyme, pepper, mustard, fennel, turmeric
Sample 12	Bay leaves	non-organic	n.s.	bay leaves
Sample 13	Bay leaves	non-organic	The Netherlands	bay leaves
Sample 14	Garam masala spice mix	non-organic	n.s.	coriander seeds, cumin seeds (22%), bell pepper, black pepper, cinnamon, coriander leaves, cardamom (4%), bay leaves, cloves, cayenne pepper
Sample 15	Italian herb mix	non-organic	n.s.	garlic, onion, bell pepper, oregano, basil, bay leaf, pepper, nutmeg
Sample 16	Bay leaves	organic	n.s.	bay leaves
Sample 17	Herb mix for fish	organic	n.s.	onion, dill, black pepper, tarragon, bay leaf, fennel seeds, thyme
Sample 18	Bay leaves	organic	Turkey	bay leaves
Sample 19	Ras el hanout spice mix	organic	n.s.	turmeric, cinnamon, fennel, clove, nutmeg, black pepper, ginger, bay leaf, cayenne pepper
Sample 20	Chimichurri dry mix	organic	n.s.	bell pepper, onion, tomato, basil, oregano, thyme, black pepper, seaweed, garlic, cumin seed, bay leaf, cayenne pepper
Sample 21	Vadouvan spice mix	organic	n.s.	onion, turmeric, cardamom, garlic, fennel seed, cumin seed, seaweed, mustard seed, carrot, tarragon, clove, fenugreek seed, cinnamon, bay leaf, cayenne pepper, lovage
Sample 22	Italian herb mix	organic	n.s.	basil, marjoram, rosemary, onion, thyme, seaweed, savory, garlic, bay leaf, bell pepper
Sample 23	Roasted vegetables herb mix	non-organic	n.s.	onion, garlic, rosemary, tomato, parsley, thyme, black pepper, bay leaf
Sample 24	Spice mix	non-organic	Sweden	black pepper, juniper (16%), roasted garlic, mushroom (5.5%), parsley, rosemary, thyme, carrot, pink pepper, coriander, bay leaf
Sample 25	Mix for beef stew	non-organic	n.s.	black pepper, mustard seed, nutmeg, onion, garlic, thyme, bay leaf

Sample Code	Product Name	Farming	Country of Production	Ingredients
Sample 26	Curry mix	non-organic	n.s.	coriander seed, turmeric, fenugreek, mustard seed, cumin seed, cinnamon, bay leaf, clove, garlic
Sample 27	Mix for chicken tikka masala	non-organic	n.s.	bell pepper, lemongrass, turmeric, cayenne pepper, coriander seeds, nutmeg, cumin seeds, fennel seeds, white pepper, garlic, onion, chives, bay leaves, thyme
Sample 28	Mix for beef stew	non-organic	n.s.	black pepper (15%), mustard seeds, nutmeg (5%), onion, thyme (5%), bay leaf (4%), garlic (5%)
Sample 29	Tajine spice blend	non-organic	n.s.	ginger, cumin (4%), cinnamon (3%), paprika, allspice, bay leaf, cardamom (1%), black pepper, carrot, onion, leek, parsnip, garlic, oregano, basil, cabbage, paprika
Sample 30	Mix for spaghetti	non-organic	n.s.	vegetables (39.9%), garlic, oregano, thyme, bay leaf, rosemary, pepper, parsley root
Sample 31	Mix for beef stew	non-organic	n.s.	onion (15%), tomato (11%), pepper, clove, parsley root, bay leaf
Sample 32	Bay leaves (fresh)	non-organic	Morocco	bay leaves
Sample 33	Bouquet garni	non-organic	n.s.	thyme, bay leaf
Sample 34	Bay leaves	non-organic	n.s.	bay leaves
Sample 35	Mix for couscous	non-organic	n.s.	red pepper, cumin, coriander seeds, pepper, dried onion, white pepper, bay leaf, clove
Sample 36	Bouquet garni for fish	non-organic	n.s.	thyme, coriander, fennel, basil, parsley, bay leaf
Sample 37	Bouquet garni for meat	non-organic	n.s.	thyme, coriander, marjoram, parsley, bay leaf
Sample 38	Bay leaves	non-organic	n.s.	bay leaves
Sample 39	Bay leaves	non-organic	Turkey	bay leaves
Sample 40	Bay leaves (crushed)	non-organic	n.s.	bay leaves
Sample 41	Mix for paella	non-organic	n.s.	dried garlic, red pepper (18%), mild pepper (17%), dried onion (14%), turmeric, bay leaf, savory, black pepper, clove
Sample 42	Bouquet garni for fish and shellfish	non-organic	EU	dried carrot (31.6%), savory, dried onion (10.5%), dried garlic, coriander seeds, black pepper, Jamaican pepper, basil, bay leaf (5.3%), fennel
Sample 43	Bouquet garni for meat and stew	non-organic	EU	savory, bay leaf (27.7%), rosemary, basil (8.2%), black pepper, thyme (4%)

Sample Code	Product Name	Farming	Country of Production	Ingredients
Sample 44	Mix for chicken	non-organic	EU	bell pepper (13%), turmeric (4%), coriander seeds (3%), onion powder, garlic powder, parsley, black pepper, thyme, ginger, bay leaf, nutmeg, cayenne pepper, cumin
Sample 45	Bay leaves (crushed)	non-organic	n.s.	bay leaves
Sample 46	Bay leaves (crushed)	non-organic	n.s.	bay leaves
Sample 47	Mix for fish	non-organic	n.s.	fennel, garlic, coriander, cumin, bay leaf, pepper, mustard, clove, Mexico pepper, fenugreek
Sample 48	Mix for chicken	non-organic	n.s.	bell pepper, coriander, turmeric, mustard seeds, cumin, chilies, fennel seeds, fenugreek, cardamom, black pepper, garlic, cloves, garlic, nutmeg, onion, chervil, chives, thyme, bay leaf
Sample 49	Bay leaves	organic	n.s.	bay leaves
Sample 50	Mix for couscous	organic	n.s.	bell pepper, coriander (22%), cumin (20%), red pepper, onion (9%), chili, bay leaf, black pepper, clove
Sample 51	Mix for paella	organic	n.s.	turmeric, dried onion, red bell pepper (14%), bell pepper, dried garlic (10%), fennel, black pepper, chili, Jamaican pepper, parsley, bay leaf (3%)
Sample 52	Bouquet garni	non-organic	n.s.	parsley, thyme, bay leaf
Sample 53	Bouquet garni	organic	n.s.	thyme, bay leaf, rosemary
Sample 54	Mix for minced meat	non-organic	n.s.	garlic, onion, paprika, oregano, basil, bay leaf, pepper, nutmeg
Sample 55	Mediterranean spices	non-organic	n.s.	rosemary (20%), thyme (5,9%), tomato (12%), onion, garlic, parsley, black pepper, bay leaf
Sample 56	Mix for mussels	non-organic	n.s.	onion, carrot, leek, tomato, parsley, thyme, celery leaves, bay leaves
Sample 57	Mix for pizza	non-organic	n.s.	oregano, cayenne pepper, red pepper, bay leaf, paprika, garlic, basil, thyme
Sample 58	Mix for fish	non-organic	n.s.	dill, fennel, bay leaf, parsley
Sample 59	Ras el hanout spice mix	non-organic	n.s.	turmeric, caraway, coriander, cumin, chili pepper, ginger, fenugreek, cinnamon, fennel, bay leaf, cloves, pepper
Sample 60	Mix for tandoori	non-organic	n.s.	bell pepper, garlic, pepper, coriander, cumin, caraway seeds, rosemary, thyme, cloves, bay leaves, cinnamon, black pepper
Sample 61	Herbes de Provence	non-organic	n.s.	bay leaf, rosemary, savory, oregano, thyme

Sample Code	Product Name	Farming	Country of Production	Ingredients
Sample 62	Mix for mussels	non-organic	n.s.	chives, parsley, tarragon, dill, bay leaf, thyme, pepper, mustard, fennel, turmeric, vegetables
Sample 63	Mix for grill	non-organic	n.s.	marjoram, basil, sage, onion, bay leaf, rosemary
Sample 64	Mix for pizza	non-organic	n.s.	oregano, chili, pepper, bay leaf, powdered paprika, garlic, basil, thyme
Sample 65	Bouquet garni	non-organic	n.s.	thyme, bay leaf, parsley

[1] n.s.: not specified.

References

1. Bylda, C.; Thiele, R.; Kobold, U.; Volmer, D.A. Simultaneous quantification of digoxin, digitoxin, and their metabolites in serum using high performance liquid chromatography-tandem mass spectrometry. *Drug Test. Anal.* **2015**, *7*, 937–946. DOI: 10.1002/dta.1781.
2. Guan, F.; Ishii, A.; Seno, H.; Watanabe-Suzuki, K.; Kumazawa, T.; Suzuki, O. Identification and quantification of cardiac glycosides in blood and urine samples by HPLC/MS/MS. *Anal. Chem.* **1999**, *71*, 4034–4043. DOI: 10.1021/ac990268c.
3. Gosetti, F.; Nebbia, C.; Ceci, L.; Carelli, G.; Marengo, E. UHPLC-MS/MS determination of oleandrin in blood and tissues of dairy cattle poisoned by oleander (Nerium oleander). *Anal. Methods* **2019**, *11*, 5562–5567. DOI: 10.1039/C9AY01800J.
4. Wang, X.; Plomley, J.B.; Newman, R.A.; Cisneros, A. LC/MS/MS analyses of an oleander extract for cancer treatment. *Anal. Chem.* **2000**, *72*, 3547–3552. DOI: 10.1021/ac991425a.
5. Wasfi, I.A.; Zorod, O.; Al katheeri, N.A.; Al Awadhi, A.M. A fatal case of oleandrin poisoning. *Forensic Sci. Int.* **2008**, *179*, e31–e36. DOI: 10.1016/j.forsciint.2008.05.002.
6. Xun, Z.; Liu, D.; Huang, R.; He, S.; Hu, D.; Guo, X.; Xian, Y. Simultaneous determination of eight alkaloids and oleandrin in herbal cosmetics by dispersive solid-phase extraction coupled with ultra high performance liquid chromatography and tandem mass spectrometry. *J. Sep. Sci.* **2017**, *40*, 1966–1973. DOI: 10.1002/jssc.201601427.
7. Yamaguchi, H.; Miyamori, K.; Sato, T.; Ogura, J.; Kobayashi, M.; Yamada, T.; Mano, N.; Iseki, K. Quantification of intracellular and extracellular digoxin and ouabain by liquid chromatography/electrospray ionization tandem mass spectrometry. *J. Chromatogr. B.* **2014**, *972*, 73–80. DOI: 10.1016/j.jchromb.2014.09.043.
8. Li, S.; Liu, G.; Jia, J.; Miao, Y.; Gu, S.; Miao, P.; Shi, X.; Wang, Y.; Yu, C. Therapeutic monitoring of serum digoxin for patients with heart failure using a rapid LC-MS/MS method. *Clin. Biochem.* **2010**, *43*, 307–313. DOI:10.1016/j.clinbiochem.2009.09.025.
9. Zhai, J.X.; Yan, H.; Shen, M.; Shen, B.H.; Liu, W. Determination of oleandrin in blood and liver samples by LC-MS/MS. *Fa Yi Xue Za Zhi* **2018**, *34*, 585–589. DOI: 10.12116/j.issn.1004-5619.2018.06.002.
10. Melo, P.; Machado, R.; Teixeira, H.M. Analysis of digoxin and metildigoxin in whole blood using solid-phase extraction and liquid chromatography tandem mass spectrometry. *Int. J. Anal. Chem.* **2012**, *2012*, 7. DOI:10.1155/2012/975824.
11. Carlier, J.; Guitton, J.; Romeuf, L.; Bévalot, F.; Boyer, B.; Fanton, L.; Gaillard, Y. Screening approach by ultra-high performance liquid chromatography-tandem mass spectrometry for the blood quantification of thirty-four toxic principles of plant origin. Application to forensic toxicology. *J. Chromatogr. B.* **2015**, *975*, 65–76. DOI: 10.1016/j.chromb.2014.10.028.
